# Natural Variation in *Arabidopsis thaliana* as a Tool for Highlighting Differential Drought Responses

**DOI:** 10.1371/journal.pone.0001705

**Published:** 2008-02-27

**Authors:** Oumaya Bouchabke, Fengqi Chang, Matthieu Simon, Roger Voisin, Georges Pelletier, Mylène Durand-Tardif

**Affiliations:** Station de Génétique et d'Amélioration des plantes, Institut Scientifique de Recherche Agronomique (INRA), Versailles, France; Max Planck Institute for Chemical Ecology, Germany

## Abstract

To test whether natural variation in Arabidopsis could be used to dissect out the genetic basis of responses to drought stress, we characterised a number of accessions. Most of the accessions belong to a core collection that was shown to maximise the genetic diversity captured for a given number of individual accessions in *Arabidopsis thaliana*. We measured total leaf area (TLA), Electrolyte Leakage (EL), Relative Water Content (RWC), and Cut Rosette Water Loss (CRWL) in control and mild water deficit conditions. A Principal Component Analysis revealed which traits explain most of the variation and showed that some accessions behave differently compared to the others in drought conditions, these included Ita-0, Cvi-0 and Shahdara. This study relied on genetic variation found naturally within the species, in which populations are assumed to be adapted to their environment. Overall, *Arabidopsis thaliana* showed interesting phenotypic variations in response to mild water deficit that can be exploited to identify genes and alleles important for this complex trait.

## Introduction

Drought is a global social and economic problem. Almost 70% of reduced yield under field conditions, compared to optimum yields in favourable cropping conditions, is due to abiotic stress [1], [2]. Understanding key mechanisms of the response to drought will help to find new levers for crop breeding. Paran and Zamir [3] listed the complex phenotypes that are controlled by similar genetic networks in different species, thus we can expect that studying the response to drought in *A. thaliana* will help to understand similar mechanisms in crops. Plant strategies to cope with drought normally involve a mixture of stress avoidance and tolerance ‘strategies’ that vary with genotype. Ludlow [4] described three general strategies plants use to cope with drought stress: dehydration tolerance, dehydration avoidance and drought escape. Dehydration tolerance refers to plants in dry environments that survive internal water deficits [5]. Dehydration avoidance involves maintaining internal water status in a dry environment by minimizing water loss and/or maximizing water uptake. Finally, drought escape is attained through a short life cycle allowing plants to reproduce before the environment becomes too dry [6], [7].

Each of these three strategies involves complex physiological mechanisms and a set of phenotypes controlled by complex gene networks. Indeed, water stress induces reversible changes in gene expression which can be studied using genomic methods such as transcription profiling. Bray previously reviewed the Arabidopsis studies [8] and categorized the induced genes into functional groups, as metabolism, transporters, signal transaction, transcription, heat-soluble hydrophilic, and unknown genes. Many drought-inducible genes have been identified by molecular and genomic analysis (reviewed by Shinozaki [9]), but increased efforts are still needed to unravel the genetic bases of plant response to drought stress as at least six signal transduction pathways exist in abiotic stress responses. Three of these pathways are known to be ABA dependent and three ABA independent but little is known about their function. Most of the studies dedicated to elucidating gene functions were carried out in *Arabidopsis thaliana,* which is now considered as an excellent model species for higher plants, even though it has no direct implications in agriculture. Numerous Arabidopsis genes involved in stress tolerance were used in genetic engineering to successfully confer drought tolerance to many different crops [10], [11], [12].

Besides genomics and mutant approaches, research based on the analysis of natural genetic variation in Arabidopsis is in full expansion [13] and has led to the to discovery of novel genes and alleles, especially in the field of plant adaptative responses to their environment [13], [14], [15].

*A. thaliana* is native to a wide range of environments with varying drought constraints, which suggests large variation in the response of these natural populations to water deficits. Genetic differences among accessions have been found in traits important in climatic adaptation to drought, e.g., flowering time and delta-^13^C [7]. Similarly, differences in water use efficiency (i.e. the ratio of dry matter gained to water lost, per unit area and per unit time) were seen between natural populations of *Boechera holboellii*, a perennial relative of *Arabidopsis thaliana* [16].

Within the collection of Arabidopsis accessions coming from natural populations, McKhann et al. [17] determined a core-collection of accessions which maximizes the genetic diversity while including a limited number of individuals. In the present study, we aimed to look for natural variation in plant response to drought within a core-collection of 24 accessions. We imposed mild water deficit conditions as it is a stress that plants frequently experience in the field [18] and measured four pertinent parameters to characterize plant response to water deficit, i.e. total leaf area (TLA), electrolyte leakage (EL), relative water content (RWC) and cut rosette water loss (CRWL). TLA was measured because leaf expansion is one of the earliest physiological signs affected by drought, much earlier than photosynthesis [19] which is usually more resilient to water deficit [20]. It was also shown that maximising the early expansion of leaf area resulted in higher crop yields in cereals growing in a water limiting context [21], [22]. The measurement of EL from plant tissue is a long-standing method for estimating membrane integrity, the degree of cell membrane stability is considered to be one of the best physiological indicators of drought stress tolerance [23], [24], [25]. The RWC reflects the plant water status [26] and CRWL is an indirect measurement of stomata aperture [27] and real transpiration of the plant (our unpublished data). Experiments on seedlings at the rosette stage indicated that some accessions show dehydration tolerance and avoidance in response to mild water deficit. We chose these interesting accessions for building promising RIL populations for future QTLs detection.

## Materials and Methods

### Plant lines

*Arabidopsis thaliana* accessions from INRA Versailles Genomic Resource Centre (http://www-ijpb.versailles.inra.fr/en/sgap/equipes/variabilite/crg/index.htm) were derived from natural populations (ecotypes) that were either prospected in the field or obtained from the Nottingham Stock Centre. Two Single Seed Descent was performed before genotyping and bulking the seed stocks for distribution. A core-collection of 24 accessions, which maximizes the amount of genetic diversity in the sample [17] while including a limited number of individuals, was used for the whole set of experiments, minus Alc-0 (178AV) and Stw-0 (92AV). In the present work we will also refer to the central core collection of 8 accessions, embedded within the core collection of 24 accessions. Col-0 (186AV) and An-1 (96AV) were systematically included as reference accessions (Table 1).

**Table 1:** Accessions included in the phenotyping experiment.

Accession	INRA Versailles Resource Centre Identification	Core collection	Country of origin
Akita	252AV	24	Japan
An-1	96AV	-	Belgium
Bl-1	42AV	24	Italy
Blh-1	180AV	8	Czechoslovakia
Bur-0	172AV	8	Ireland
Can-0	163AV	16	Spain
Col-0	186AV	-	Poland
Ct-1	162AV	8	Italy
Cvi-0	166AV	8	Cape Verde
Edi-0	83AV	24	Scotland
Ge-0	101AV	16	Switzerland
Gre-0	200AV	24	USA
Ita-0	157AV	8	Morocco
Jea	25AV	8	France
Kn-0	70AV	24	Lithuania
Mh-1	215AV	16	Poland
Mt-0	94AV	16	Libya
N13	266AV	16	Russia
Oy-0	224AV	8	Norway
Pyl-1	8AV	16	France
Sakata	257AV	24	Japan
Shahdara	236AV	8	Tadjikistan
St-0	62AV	16	Sweden
Tsu-0	91AV	24	Japan

The first column shows the common accession name. The second column refers to the identification number in the INRA Versailles Resource Centre database (http://dbsgap.versailles.inra.fr/vnat/). The number in the third column indicates the rank of the core-collection according to McKhann *et al*., 2004. The fourth column indicates the country in which the accession was collected, according to the information available at the International Stock Centre.

### Growth conditions and drought stress

All experiments were performed in Fertiss© substrate in 4×4 cm clods (70% blond peat, 20% perlite and 10% vermiculite). Five samples of non-perturbed clods were used to determine a water retention curve (WRC) i.e. the relationship between soil volumetric water content and soil suction expressed as log value (pF), with the help of a membrane pressure apparatus [28] model 1500. Watering volumes were determined on this relationship for both control and mild water deficit treatments. Soil water content was fixed at 60% of substrate maximal water content (pF = 1,3) as a control. At this value, none of the measured parameters was affected compared with saturated irrigation (not shown). The water-deficit treatment was fixed at 30% of the substrate maximal water content (pF = 3,2). Clods were weighed daily and adjusted to a fixed weight corresponding to the water contents described above. The two watering regimes were applied at the emergence of leaf number 6.

Seeds were stratified in a 0.1% agar solution for 3 days at 4°C in the dark before being sown on watered clods. The substrate was irrigated with 0.5× Hoagland's nutrient solution (i.e. K^+^, 2.6 mE; Ca^2+^, 3.1 mE; Mg^2+^, 0.8 mE; NH_4_^+^, 0.9 mE; H^+^, 1.8 mE ; NO_3_^−^, 5.1 mE ; PO_4_H_2_^−^, 0.9 mE ; SO_4_^2−^, 0.9 mE) from the sowing date until the 6th leaf stage. Plantlets were only kept for the experiments if their leaf number was homogeneous compared to that of the whole experimental set and their rosette size was within the range of a given genotype.

Plants were grown in a growth chamber under short days (8h light/16h dark), with a mean air temperature of 21°C and the relative humidity never decreased below 60%. In order to minimize the position effect in the chamber, trays containing plants (5×6) were rotated daily back to front and right to left.

Plants were collected for Electrolyte Leakage (EL), Relative Water Content (RWC) and Cut Rosette Water Loss (CRWL), after 7 days of fully watered or water deficit treatment and after 10 days for total leaf area (TLA) measurement.

### Measurements

- Total leaf area (TLA, cm^2^): pictures of the canopy were taken of each plant. TLA was obtained with the Optimass© software thanks to a macro written by P. Belluomo (INRA Grignon). 4 plants per genotype and per treatment were used for each measurement.
- Electrolyte Leakage (EL, %) was used to evaluate cell membrane integrity by measuring the electric conductivity. The method was adapted from Liu et al. [29]. Rosettes were cut, placed in 10ml-deionised water and shaken for 20 minutes. Conductivity (C_A_) of the solution was measured with a conductimeter. The solution was then boiled for 25 minutes, cooled at room temperature and conductivity measured again (C_B_). The EL was calculated using the formula: (C_A_/C_B_)*100. 3 plants per genotype and per treatment were used to measure the EL.
- Relative Water Content (RWC, %) was calculated according to the formula: (FW-DW)/(TW-DW). Fresh weight (FW) was obtained by harvesting and weighing freshly detached rosettes. Turgid weight (TW) was obtained by putting cut rosettes into a tube with de-ionized water for 16 hours at room temperature, removing excess water by wiping with absorbent paper and weighing plant material. Rosette dry weight (DW) was recorded after an overnight incubation at 75°C in a dry oven.
- Cut Rosette Water Loss (CRWL,%), indicating the amount of water lost from freshly cut tissues in the first two hours, was obtained according to the method described by Lefebvre et al. [30]. Freshly cut rosettes were harvested and weighted (FW). Rosettes were maintained in the growth chamber environmental conditions then weighted after 2 hours (W_2_), then left overnight at 75°C in a dry oven. Rosette dry weight was recorded (DW). CRWL was calculated according to the formula: (FW-W_2_)/(FW-DW)*100.

The dataset is compiled in Supplementary information (Table S1). Means and Standard Deviations are given in Supplementary information (Table S2).

### Statistical analysis

For Principal Component Analysis and Classification, the normality of the data was improved using logarithmic and Gaussian transformations with a mean of 0 and a variance of 1. The classification method used hierarchical cluster algorithms (Upgma or arithmetic mean method). GenANOVA software was used for PCA and classification [31]. Other statistical analyses, including analysis of variance (ANOVA) were carried out with STATGRAPHICS Plus 5.0 software.

## Results

### Mild water deficit significantly affected physiological traits revealing genetic variation among the 24 studied accessions

Even though water deficit conditions were mild and applied for a short period, these had a significant effect (P<0.001) on the four parameters measured. Physiological responses varied between accessions and a significant genotype×environment interaction was observed (Table 2).

**Table 2:** Two-factors ANOVA table

	Electrolyte Leakage		Relative Water Content		Total Leaf Area		Cut Rosette Water Loss	
Main effects	F-Ratio	P-Value	F-Ratio	P-Value	F-Ratio	P-Value	F-Ratio	P-Value
Water treatment (WT) ddl = 1	199,2	***	96,5	***	274,67	***	63,54	***
Accession (A) ddl = 23	14,44	***	14,81	***	26,97	***	18,97	***
Interaction WtxA ddl = 23	7,14	***	5,66	***	3,33	***	2,34	**

*** Significant at P<0,001

** Significant at P<0,01

ANOVA has been run on the whole set of accessions, showing the effect of “Water Treatment” and “Accession” factors on the 4 measured parameters: Electrolyte Leakage, Relative Water Content, Cut Rosette Water Loss and Total Leaf Area.

**Figure 1:**
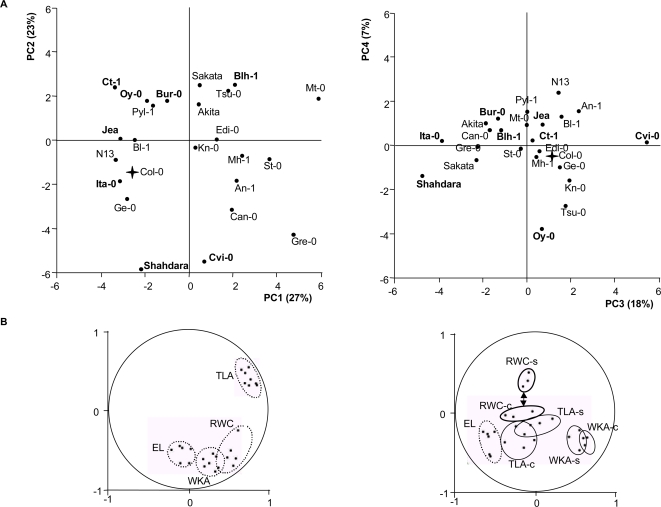
A Principal Component Analysis of control and stressed plants. The 8 accessions from the central core-collection are in bold type. The Col-0 accession is indicated by a cross. 1A/Repartition of accessions on the first (PC1 and PC2 axis) and second PCA planes (PC3 and PC4 axis). PC1 explains 27% of the variation, PC2: 23%, PC3: 18% and PC4:7%. 1B/Plots of the first and the second PCA planes on correlation circles. The measured characters are indicated respectively for watered (c) and stress (s) plants: TLA = Total leaf area, EL = Electrolytes Leakage, RWC = Relative Water Content, WKA = Water Keeping Ability. TLAc and TLAs, WKAc and WKAs, RWCc and RWCs and MDRc and MDRs are plotted on the same area, on the circle of correlation corresponding to the first plane. The MDRc and MDRs are plotted on the same area, on the circle of correlation corresponding to the second plane.

In order to group individuals according to their response to water deficit, we performed a global Principal Component Analysis (PCA) on the plants that were watered with and without restriction. Fig. 1A, shows the first factorial plane delimited by the PC1 and PC2 axes, which accounted for 27 and 23% of the observed variation respectively. As shown in Fig. 1B, by the correlation circle , the first axis is essentially explained by Total Leaf Area (TLA) under watered or water deficit conditions and to a lesser extent by Relative Water Content (RWC). Cvi-0, Shahdara, Gre-0 and Mt-0 are located far from the origin of the axis on Fig. 1A, suggesting that these plants reacted differently to the average of the studied accessions. The correlation circle generated by PC1 and PC2 shows that traits under watered or stress conditions are located close-by, meaning that the quantitatively important variation (27% for PC1 and 23% for PC2), which is 50%, is on the same relative value for accessions in watered and water deficit conditions: globally, accessions showing a large rosette in watered conditions, still had a relative large rosette in stress conditions. On the second plane, explaining 26% of the variation, outliers are Cvi-0, Oy-0 and Shahdara from the central core-collection and, to a lesser extent, Ita-0 and Tsu-0 from the core-collection. The correlation circle showed that, except for Electrolyte Leakage (EL), there is a distinct separation of parameters measured in watered and stress conditions. The parameter which showed the largest contrast in this regard is RWC which was highly significant in the PC4 axis for the stressed plants but almost insignificant for the control plants, indicating that RWC does not vary considerably among accessions in the absence of water deficit.

### Plant response to water deficit cannot be predicted by observing its phenotype in watered conditions

**Figure 2:**
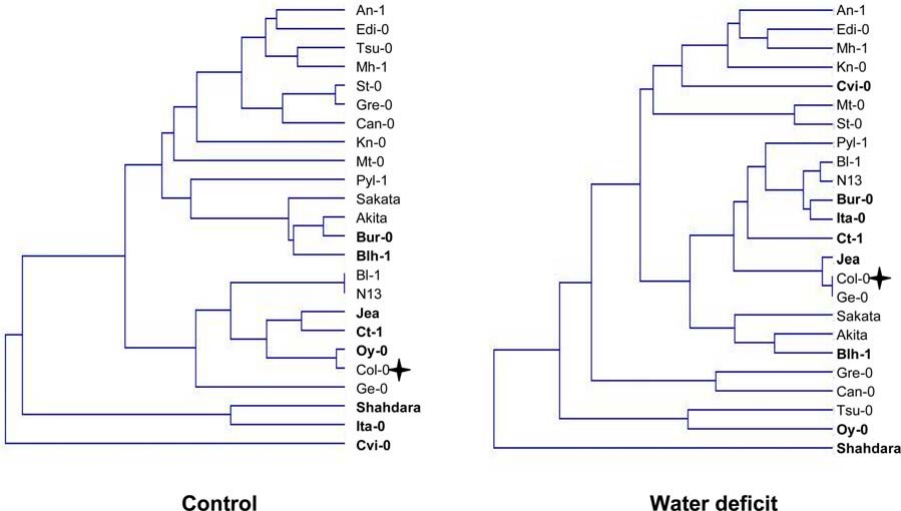
Dendrogram of the upgma classification of the whole set of accessions, under watered and water deficit conditions. The central core-collection accessions are in bold. The Col-0 accession is indicated by a cross.

Next, we classified the accessions using cluster algorithms of the data obtained in the control and water deficit conditions (fig. 2). In the dendrogram of accessions grown under control conditions, three accessions from the central core-collection, Cvi-0, Ita-0 and Shahdara, are seen to form a distinctly separate group. Adding to that, the accessions of the central core-collection are grouped together on one side of the tree. The branching pattern is different for the dendrogram of accessions grown under stress conditions. This suggests that the response to drought of a particular accession cannot be predicted by observing its phenotype under watered conditions, which shows extended variation. The 8 accessions from the central core-collection are much more spread out on the tree. Specifically, the Shahdara accession is furthest away from the main group, Cvi-0 is now close to accessions that were far from it on the tree of the control plants and Ita-0 has a phenotype similar to Bur-0 in stress conditions while the latter two accessions are not very different from N13 and Bl-1.

### Some accessions followed different dehydration tolerance or avoidance scenarios in their response to water deficit

**Figure 3:**
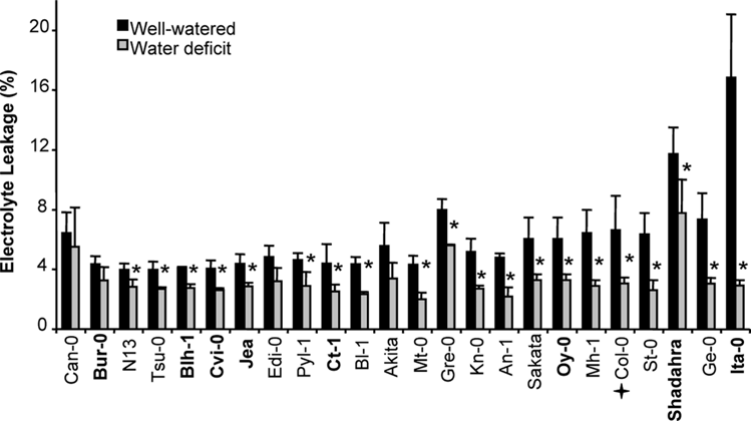
Electrolyte Leakage (EL,%) in rosettes of 24 accessions of *Arabidopsis thaliana*, in watered (black bars) and water deficit (grey bars) conditions. Vertical bars are SE at the 95% confidence level (n = 3). Accessions are ordered on the X-axis, from left to right, according to increased reduction in EL following water deficit. The central core-collection accessions are in bold. Col-0 is indicated by a cross. * indicates a statistically significant difference between control and water deficit conditions at 95% confidence level, determined with a non parametric test (Kruskall-Wallis).

**Figure 4:**
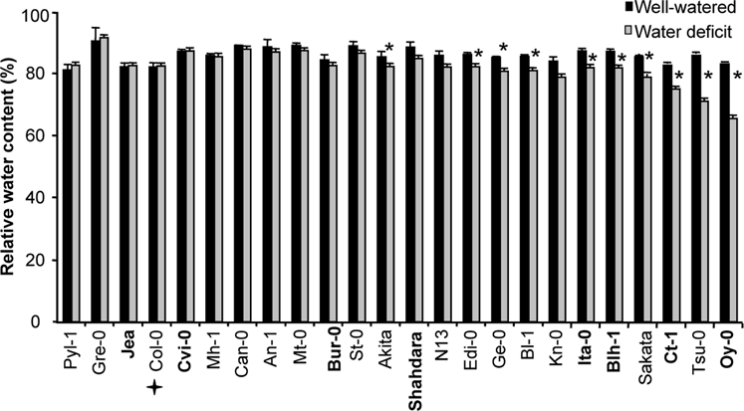
Relative Water Content (RWC,%) in rosettes of 24 accessions of *Arabidopsis thaliana*, in watered (black bars) and water deficit (grey bars) conditions. Vertical bars are SE at the 95% confidence level (n = 3). Accessions are ordered on the X-axis, from left to right, according to increased reduction in RWC following water deficit. The central core-collection accessions are in bold. Col-0 is indicated by a cross. * indicates a statistically significant difference between control and water deficit conditions at 95% confidence level, determined with a non parametric test (Kruskall-Wallis).

**Figure 5:**
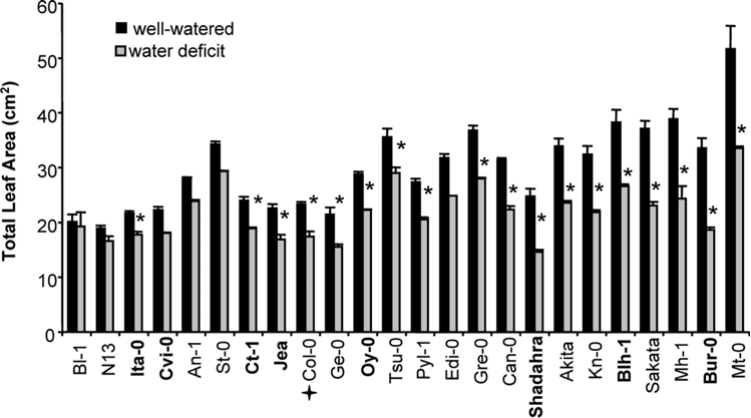
Rosettes Total Leaf Area (TLA, cm^2^) of 24 accessions of *Arabidopsis thaliana*, in watered (black bars) and water deficit (grey bars) conditions. Vertical bars are SE at the 95% confidence level (n = 4). Accessions are ordered on the X-axis, from left to right, according to increased reduction in TLA following water deficit. The central core-collection accessions are in bold. Col-0 is indicated by a cross. * indicates a statistically significant difference between control and water deficit conditions at 95% confidence level, determined with a non parametric test (Kruskall-Wallis).

**Figure 6:**
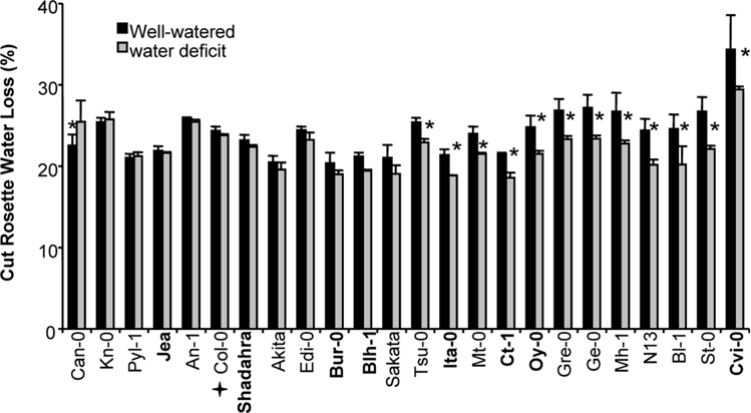
Cut Rosette Water Loss (CRWL, %) of 24 accessions of *Arabidopsis thaliana*, in watered (black bars) and water deficit (grey bars) conditions. Vertical bars are SE at the 95% confidence level (n = 3). Accessions are ordered on the X-axis, from left to right, according to increased reduction of CRWL following water deficit. The central core-collection accessions are in bold. Col-0 is indicated by a cross. * indicates a statistically significant difference between control and water deficit conditions at 95% confidence level, determined with a non parametric test (Kruskall-Wallis).

After an overall assessment of the accessions response to water deficit, we looked more closely at each parameter. Surprisingly, as seen in fig. 3, a systematic lower level of electrolyte leakage was observed for all accessions following soil desiccation. This decrease in EL in drought compared to control conditions was more pronounced for Ita-0, St-0 and Ge-0 but not very obvious for Can-0, Bur-0, N13 and Gre-0. RWC varied moderately between accessions in control conditions (Fig. 4) and overall was not highly affected by water treatment. Nevertheless, for some accessions such as Oy-0 and Tsu-0, RWC decreased significantly, while for some others, such as Cvi-0, Col-0, Jea, RWC did not vary at all, or even increased compared to the control (Gre-0, Pyl-1). TLA was the most responsive trait among accessions in control and water deficit conditions (fig. 5). The accessions with the greatest reduction in TLA in response to water deficit were Bur-0, Shahdara, Sakata, Mh-1, and Mt-0. The TLA of other accessions such as Bl-1, N13, St-0 and An-1 was less affected. Finally, Cut Rosette Water Loss (fig. 6) varied among accessions. Cvi-0 showed the greatest level of water loss in control and stress conditions. Bl-1, N-13 and St-0 showed the largest decrease in CRWL in response to water deficit. For other accessions: Kn-0, Pyl-1, Jea, An-1, Col-0 and Shahdara, water loss was almost at the same level as in control conditions. CRWL was higher in water deficit conditions for Can-0.

## Discussion

### Arabidopsis showed substantial natural genetic variation in response to water deficit

The *Arabidopsis* accessions studied here originated from different geographical regions (tab.1). Considering the heterogeneous distribution of ground water and precipitations across the globe, different natural populations were potentially subjected to different selective pressures. In these conditions we would expect notable variations in the accessions' response to water deficit. Indeed, a significant genotype×environment interaction was observed for each of the parameters measured. Thus, accessions may have developed an adaptive response to drought that can be exploited to further determine the genetic variability responsible for this physiological adaptation.

The PCA showed that overall the accessions from the core-collection display a balanced range of observed phenotypes. McKhann *et al.* [17] already showed that the optimised collection of 24 accessions encompasses most of the morphological diversity present among Arabidopsis accessions, and that a larger core-collection only leads to higher levels of redundancy rather than novelty. Following our PCA and classification of the accessions merging all the parameters measured in both watered and drought conditions, several accessions appear to be good candidates for further studies which may reveal unknown major genes and alleles for responses to drought. Among these accessions are Shahdara and Cvi-0.

Previously, the An-1 accession was reported to be drought-tolerant [32], [33]. In this study, we observed that this accession, based on all the parameters measured with the exception of electrolyte leakage, was hardly affected by the mild drought conditions. Compared to other accessions, it maintained its water status because tissue RWC remained almost the same in control and water deficit conditions and water loss from freshly cut rosettes was significantly less affected than in other accessions. Furthermore, the TLA of An-1 in water deficit conditions was almost 85% of its control TLA. As a comparison, the TLA of Bur-0 plants, which were the most severely affected by water stress was only 55% of its control TLA, and the least affected accession, Bl-1, maintained 95% of TLA compared to plants in control conditions.

Other accessions showing drought tolerance such as Oy-0 and Tsu-0 or drought avoidance such as Mh-1 are also very interesting potential candidates in genetic programmes aimed at discovering new genes involved in plant response to water deficit. If dehydration tolerance is considered as the ability to survive internal water deficits, Oy-0 and Tsu-0 showed tolerance ability as these accessions maintained their growth even though their water status i.e. RWC, was strongly affected.

Maximizing growth rate despite a reduction in water status is a tempting strategy. But it is also water consuming (by increasing the area transpiring) if the drought cycle is very long. In this case, drought avoidance appears to be a better strategy for saving water throughout the plant growth cycle. Indeed, both scenarios (tolerance and avoidance) are important drought adaptation strategies that could be used in breeding programmes depending on the environmental conditions targeted and drought status.

Some accessions showed dehydration avoidance by minimizing water loss, i.e. minimizing TLA and/or CRWL. Mh-1 displayed both reduced leaf area and tissue water loss by stomatal closure. Shahdara's leaf area decreased significantly but water loss was only very slightly reduced, while the group of accessions, Bl-1, N13 and St-0, responded inversely by maintaining TLA and reducing CRWL. The accessions Pyl-1, Can-0, An-1, Jea and Col-0 showed an atypical reaction by maintaining almost the same levels of CRWL and RWC between control and water deficit conditions.

It is striking that a systematic lower level of electrolyte leakage was observed for all accessions following soil desiccation, which may indicate improved membrane integrity in mild drought conditions probably due to membrane hardening. Similar results were observed in resurrection plant by Quartacci et al. [34] where a membrane injury index decreased with dessication. This decrease was accompanied by an enrichment in free sterols which was interpreted as a mechanism of drought adaptation based on sterol-induced membrane rigidification.

Finally, Col-0, the reference accession, displayed a standard average response to both watered and water deficit conditions. The PCA grouped it with most of the other accessions in both control and water deficit conditions.

### Outliers can be used to build new RIL sets which can be exploited to find new genes involved in water stress response

When building a RIL population, the choice of the parents is a key point that determines successful QTL detection and gene mapping. Choosing extremes of the genotypic variation within a species increases the likelihood of obtaining a valid RIL population showing segregation and transgression for the studied trait and for identifying QTLs with a high probability (LOD score).

LerxCvi recombinant lines have already been widely used for QTLs identification for diverse characteristics, such as tolerance to biotic and abiotic factors, developmental traits i.e. flowering time, physiological traits, enzymatic activities…(reviewed by Koornneef *et al*. [35]). QTLs for carbon isotope discrimination delta-^13^C, were identified by Juenger *et al.* [36] within this same RIL population. In C_3_ plants like *A. thaliana*, delta-^13^C is correlated with one of the targets for plant drought adaptation, namely Water Use Efficiency, i.e. the carbon unit gain per water unit loss, as a result of a balance between stomatal conductance and photosynthetic activity.

The RIL set Bay-0xShahdara generated by Loudet et al. [37] has also been extensively used with success to detect QTLs for diverse traits, as illustrated by recent publications: senescence [38], primary cell wall composition [39], response to phosphate starvation [40], starch and sugar content [41], resistance to *Pseudomonas syringae* [42] and expression QTLs for gene networks [43].

The INRA Versailles Resources Centre for Genomics is currently producing large RIL populations from crosses between Col-0, as the male parent, and each of the 8 accessions from the central core-collection (Simon M. *et al*., in preparation). In this study, this collection showed a very promising variation in phenotypic traits related to drought. Of specific interest will be the RIL populations generated with the Cvi-0 and Shahdara accessions, which were highlighted in our PCA and are now available including a complete genetic map. RIL populations with Bur-0, which showed a strongly affected TLA, and Oy-0 which appears drought tolerant, will also be available soon. Adding An-1 as a parent will certainly contribute to further understanding the genetic basis of drought tolerance in Arabidopsis.

In conclusion, we have shown that Arabidopsis thaliana displays variation in its response to drought that can be exploited to find genes and alleles important for this complex character. Arabidopsis thaliana has been largely adopted for mendelian genetics investigations and genomics but it is also a promising species for the pursuit of quantitative genetics studies, as already proven by the isolation of genes underlying QTLs [44].

## Supporting Information

Table S1 Experimental data. Measures performed on each individual plant are indicated: Electrolyte Leakage (%), Relative Water Content (%), Water Keeping Ability (%), Total Leaf Area (cm2), for control and stress treatment. Accessions are identified with their identification number in the INRA Versailles Resource Centre for Genomics (http://dbsgap.versailles.inra.fr/vnat/) and their common name.
(0.03 MB XLS)

Table S2 Means and Standard Deviations. Means and Standard Deviations are indicated for each character measured: Electrolyte Leakage (%), Relative Water Content (%), Water Keeping Ability (%), Total Leaf Area (cm2), for control and stress treatment. Accessions are identified with their identification number in the INRA Versailles Resource Centre for Genomics (http://dbsgap.versailles.inra.fr/vnat/) and their common name.
(0.03 MB XLS)
